# Assessment of gut microbial β-glucuronidase and β-glucosidase activity in women with polycystic ovary syndrome

**DOI:** 10.1038/s41598-023-39168-5

**Published:** 2023-07-24

**Authors:** Jalpa Patel, Hiral Chaudhary, Kiransinh Rajput, Bhavin Parekh, Rushikesh Joshi

**Affiliations:** 1 Department of Biochemistry and Forensic Science, University School of Sciences, Gujarat University, Ahmedabad, Gujarat 380009 India; 2 Department of Microbiology and Biotechnology, University School of Sciences, Gujarat University, Ahmedabad, Gujarat 380009 India; 3 School of Applied Sciences and Technology, Gujarat Technological University, Ahmedabad, Gujarat 380009 India

**Keywords:** Endocrine reproductive disorders, Endocrine system and metabolic diseases

## Abstract

PCOS is a prevalent endocrine disorder among women of reproductive age, characterized by hormonal imbalances and metabolic disturbances. This study explores the correlation between gut microbial β-glucuronidase and β-glucosidase and PCOS, focusing on their association with hormone levels and other clinical parameters. In this case-control study, fecal samples were collected from women of reproductive age, both with and without PCOS. The analysis of gut β-glucuronidase and β-glucosidase enzymes was conducted with the other clinical parameters, including body mass index, hormone levels, and hirsutism. These factors were then subjected to correlation analysis. PCOS women showed significantly higher levels of β-glucuronidase activity with a statistically significant *P*-value (0.05 ± 0.1 vs. 0.04 ± 0.1; *P* = 0.006) as well as β-glucosidase activity (0.13 ± 0.08 vs. 0.09 ± 0.05; *P* = 0.06) compared to the controls. This study also revealed intriguing connections between the selected enzymes and hormone levels, particularly testosterone and estradiol. Gut microbial enzymes β-glucuronidase and β-glucosidase may be used as biomarkers for early detection and monitoring of PCOS in women with metabolic challenges. It could lead to improved diagnostic tools and treatment options.

## Introduction

Polycystic ovarian syndrome (PCOS) is a common endocrine condition in women of reproductive age, with an estimated worldwide prevalence of 8% to 13%. PCOS is the leading cause of female infertility^1^. PCOS is characterized by polycystic ovaries, chronic anovulation, hyperandrogenism, hyperinsulinemia, abdominal obesity, and dyslipidemia, which can lead to serious long-term issues such as endometrial hyperplasia, type 2 diabetes, and coronary artery disease^2,3^. The pathogenesis of PCOS remains nebulous. However, it is considered multifaceted, involving genetics, the intrauterine environment, and lifestyle choices^4^.

The gut microbiota functions as an endocrine organ that can affect other distant organs^5^. Using metagenomics tools, researchers have shown that many chronic conditions, such as obesity, diabetes, non-alcoholic fatty liver disease (NAFLD), and PCOS, are all linked to gut microbial dysbiosis^6^. Likewise, the role of gut microbes has recently emerged as an exciting research area in PCOS pathology^7^. Since 2017, more than a dozen clinical cross-sectional studies have shown that PCOS correlates with gut dysbiosis, while the taxa vary among these studies. Most studies have shown reduced α-diversity and β-diversity, both of which are reported to be associated with endocrine and metabolic abnormalities^8^.

Most of these cross-sectional studies have used 16S rRNA gene amplicon sequencing. Although 16S rRNA sequencing provides accurate taxonomic resolution down to the genus level, it cannot measure specific bacterial functions, which may better indicate how bacteria affect PCOS pathology^9^. PCOS-specific bacterial functions remain scantly explored as characterized by microbial enzymatic activity.

Since gut microbial β- glucuronidase and β-glucosidase enzymes, found in various species of gut bacteria, mediate hormonal (i.e., estrogen) and metabolic homeostasis^10^, we wondered whether levels of these enzymes differ between Polycystic ovary syndrome (PCOS) women and healthy women.

This is the first study to evaluate the activity of β-glucuronidase and β-glucosidase in stool specimens of women with and without PCOS. The gut microbial enzyme activity was measured in triplicate from the same stool specimen collected independently by each participant. Herein, we measured and correlated the enzyme activities with clinical parameters including hormones.

## Materials and methods

### Participants and ethics

Forty-eight women were recruited, 23 with PCOS and 25 healthy (Fig S1). These women, aged 17–42 years, were enrolled individually who visited the Health Centre, Gujarat University (Gujarat, India) during December, 2019 to December, 2022. Diagnosis criteria for PCOS, established by the Health Centre of the Institute, were based on the Rotterdam Criteria and the currently available evidence in Indian women. In our study, to avoid confusion due to the different diagnostic criteria, each participant had met two out of three features based on Rotterdam criteria as follows: Oligo- or anovulation, signs of hyperandrogenism (clinical and biochemical), and polycystic ovaries (PCO, at least 12 follicles measuring 2–9 mm or volume of the ovary > 10 cm^3^) (Rotterdam ESHRE/ASRM- Sponsored PCOS Consensus Workshop Group, 2004). The control women group had no history of diagnosed PCOS.

We recorded multiple clinical parameters, including medical history, dietary habits, and family history. All subjects received a standard physical examination of height, weight, waist circumference, and hip circumference in light clothing without shoes. BMI and waist-to-hip ratio (WHR) were calculated. Present study was endorsed by the Ethical Committee of Gujarat University (GU-IEC(NIV)/02/Ph.D./007) and all research was performed in accordance with relevant guidelines and regulations. All participants were verbally explained about the research plan and asked to sign the bilingual (English and Gujarati) consent form and then after where enrolled for proposed work.

### Inclusion and exclusion criteria

The inclusion criteria for this study were (a) 17–45 years of age and (b) Indigenous ethnicity. In addition, individuals with one of the following conditions have been excluded from the study: pregnancy, hypertension, smoking, thyroid dysfunction, Cushing’s syndrome, adrenal disorders, hyperprolactinemia, gastrointestinal disease, and Diabetes mellitus. Participants also had no administration of hormonal medication, an insulin sensitizer, or antibiotics within the preceding three months.

### Laboratory measurements

Baseline fasting blood samples were taken on 2–5 days of the menstrual cycle for hormonal and metabolic measurements. The serum aliquots have been stored at -80˚C until analysis. We measured levels of follicle-stimulating hormone (FSH), luteinizing hormone (LH), total testosterone, fasting blood glucose, estradiol, thyroid-stimulating hormone (TSH), and dehydroepiandrosterone sulfate (DHEAS) were measured.

All participants were given a brief questionnaire to collect general information on demographics, health, medication use, lifestyle-eating habits, and illness conditions that may affect the gut microbiota and alter gut bacterial enzyme activity.

### Fecal specimen collection

After consent, participants were asked to collect fecal samples at home. They were supplied specimen collection kits, including a freezer pack and a barcoded container with exemplified instructions for collecting specimens. After fecal collection, the fresh samples were transported directly to our laboratories on pre-frozen freezer packs within two hours and stored at − 80 °C until analysis.

### Protein extraction

Protein extraction and enzymatic activities were carried out as described by Flores et al. with slight modifications to optimize the detection of the enzymatic activities^11^. Approximately 0.5 gm of thawed fecal material in 5 ml of phosphate-buffered saline (PBS) was transferred to a 10 mL conical tube containing 5 mL of extraction buffer (60 mM Na_2_HPO_4_, 40 mM NaH_2_PO_4_, 10 mM Kcl, 1 mM MgSO_4_) and kept on ice. Fecal material was treated by heavy vortex for 1 min, and bacterial cells were lysed by sonication at maximum power for 90 s (30 s intervals) on an ice bath. Lysates were centrifuged at 4 °C, 7000 rpm for 30 min. The supernatant containing extracted proteins was transferred to new tubes and was used to measure protein concentration and enzymatic activities. Protein concentration in each lysate was estimated using the Folin Lowry method.

### Enzyme assay

**Figure 1:**
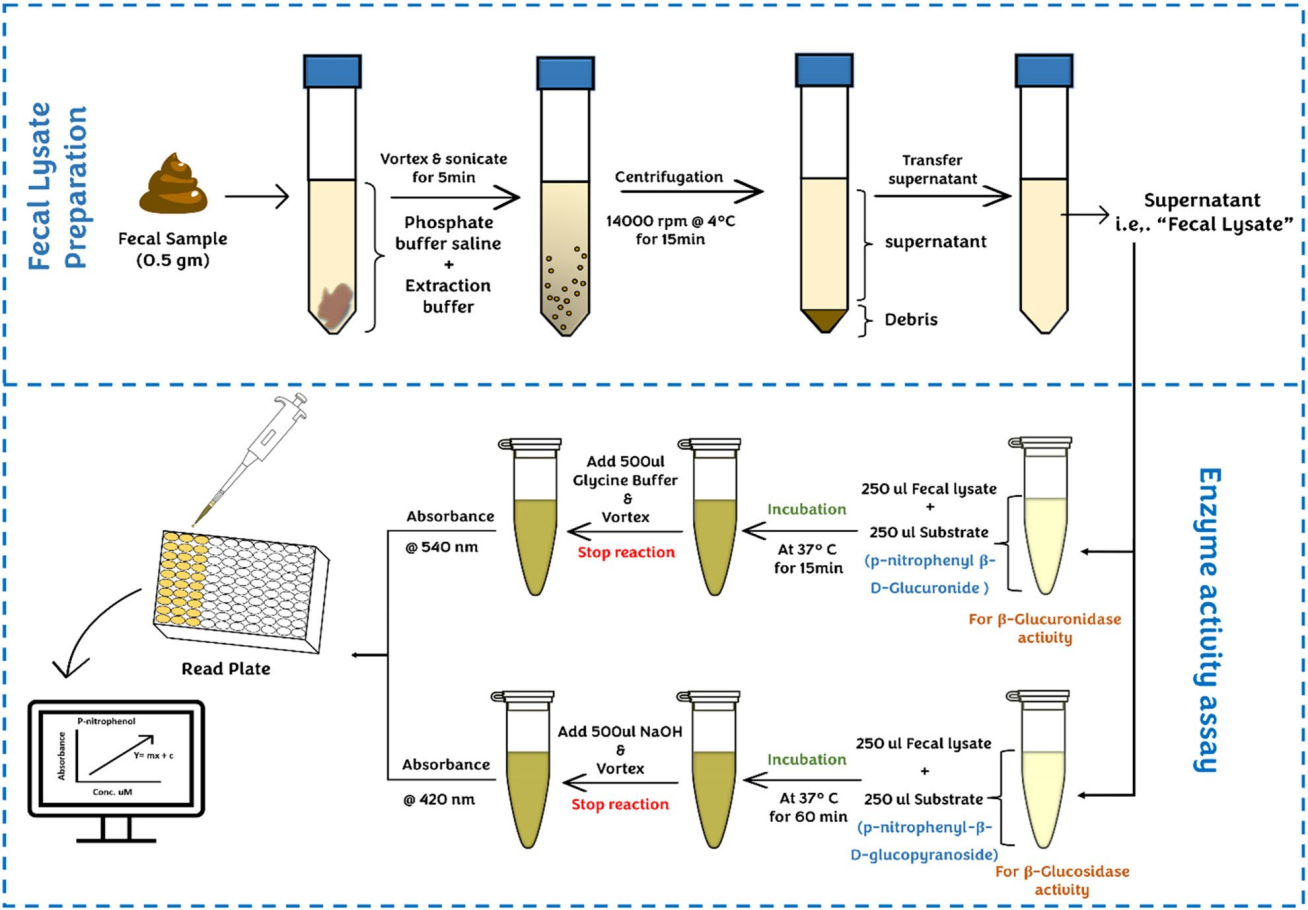
Method overview of fecal lysate preparation and enzyme activity assay. Fresh-frozen or freshly collected fecal pellets were weighed, followed by vortex and sonicated in a mixture of phosphate buffer saline pH 7.4 and extraction buffer. Following centrifugation, the supernatant was isolated (fecal lysate) and used in a colorimetric enzymatic assay. The fecal lysate was divided into two tubes, and an enzyme assay was done for β-glucuronidase and β-glucosidase.

Gut microbial enzyme activity was measured as follows: the reaction mixture (total volume of 0.5 ml) contained 0.25 ml of 2 mM p-nitrophenyl β-D-Glucuronide or/and 2 mM *p*-nitrophenyl-β-D-glucopyranoside for β-D-glucuronidase and β-glucosidase respectively, and 0.25 ml of fecal suspension. The assay mixture was incubated at 37 c for 15 min for β-glucuronidase and 60 min for β-glucosidase. The reaction was quenched by adding 0.5 ml of 80 mM of glycine buffer pH 10 and 0.5 N NaOH for β-glucuronidase and β-glucosidase, respectively, and vortex it. The absorbance of the product of both enzymes, i.e., *p*-nitrophenol, was measured at 540 nm and 420 nm for β-glucuronidase and β-glucosidase, respectively (Fig. 1).

The activity of β-glucuronidase and β-glucosidase was assessed through end-point spectrophotometric assays, measuring the p-nitrophenol released from the hydrolysis of their respective substrates. A meticulously prepared standard calibration curve was employed to ensure accurate quantification of *p*-nitrophenol (0.025 mM to 0.25 mM), serving as a reference for evaluating sample values.

A unit of enzyme activity corresponds to the quantity of *p*-nitrophenol, presented in mM, released during a one-hour reaction for each milligram of protein. The methodology implemented was particularly suitable for our study design's single-time stool sample collection.

The overall data, expressed as mean values with their standard deviation, encompasses at least three independent experiments. For comprehensive details on preparing the standard curve, please refer to the supplementary files (Fig S2).

### Statistical analysis

The software program GraphPad Prism 9 was used to process experimental data. The data are shown as mean ± SD. All the experimental data were normally distributed or transformed to normal distribution. The statistical difference across the groups was assessed using a one-way ANOVA, t-test, and chi-square test, and *p* ˂ 0.05 was considered significant.

Pearson’s correlation was used to evaluate the relationship between the two enzymes’ activities. The relationship between the two enzymatic activity levels and Clinical parameters was estimated with Pearson’s correlation. Significance was based on trend or two-sided categorical tests with α = 0.05.

### Ethical approval

The Institutional Ethics Committee (IEC) of the University School of Sciences, Gujarat University, approved the study with reference ID: GU-IEC(NIV)/02/Ph.D./007.

## Result

### Demographic, clinical, and biochemical parameters of the study subjects

The clinical characteristics of the recruited participants are summarized in Table 1. PCOS and the controls were matched for age and BMI. Compared to the control group, women with PCOS showed significantly higher levels of testosterone (*p *˂ 0.05), estradiol (*p *˂ 0.05), LH/FSH ratio (*p *˂ 0.05, and hirsutism score (*p *˂ 0.05). The levels of TSH, LH, prolactin, and DHEAS were not different among the groups.

**Table 1:** Demographic and clinical parameters of study participants.

Variables and parameters	Control group	PCOS group	*P* value
Demographic variables and Clinical features			
N	25	23	**–**
Age (year)	27.47 ± 5.8	27 ± 6	**–**
Body Mass Index (kg/ m^2^)	24.76 ± 5.6	24.98 ± 5.15	> 0.05
Waist-to-hip ratio	0.77 ± 0.05	0.78 ± 0.07	> 0.05
LH (mIU/ml)	5.22 ± 3.1	6.30 ± 4.3	> 0.05
FSH (mIU/ml)	11.29 ± 7.8	7.99 ± 2.5	**0.05**
LH/FSH	0.55 ± 0.29	0.84 ± 0.73	**0.04**
PRL (ng/ml)	19.14 ± 9.74	26.84 ± 32.71	> 0.05
T (ng/dl)	14.67 ± 4.9	34.56 ± 36.08	**0.01**
E2 (pg/ml)	84.09 ± 18.04	69.38 ± 23.24	**0.01**
TSH (uIU/ml)	2.17 ± 1.4	1.8 ± 0.99	> 0.05
DHEAS (ug/dl)	143.56 ± 50.04	166. 17 ± 105.05	> 0.05
Hirsutism (Ferriman-Gallwey)	5.91 ± 2.8	9.65 ± 5.9	**0.001**
Gut bacterial enzyme activity			
Total Protein (mg/ml)	1.35 ± 0.28	1.43 ± 0.31	> 0.05
β-glucuronidase (mM/mg/hr)	0.04 ± 0.01	0.05 ± 0.01	**0.01**
β-glucosidase (mM/mg/hr)	0.09 ± 0.05	0.13 ± 0.08	> 0.05

### Gut microbial β-glucuronidase and β-glucosidase enzyme activity differed between PCOS and control groups

**Figure 2:**
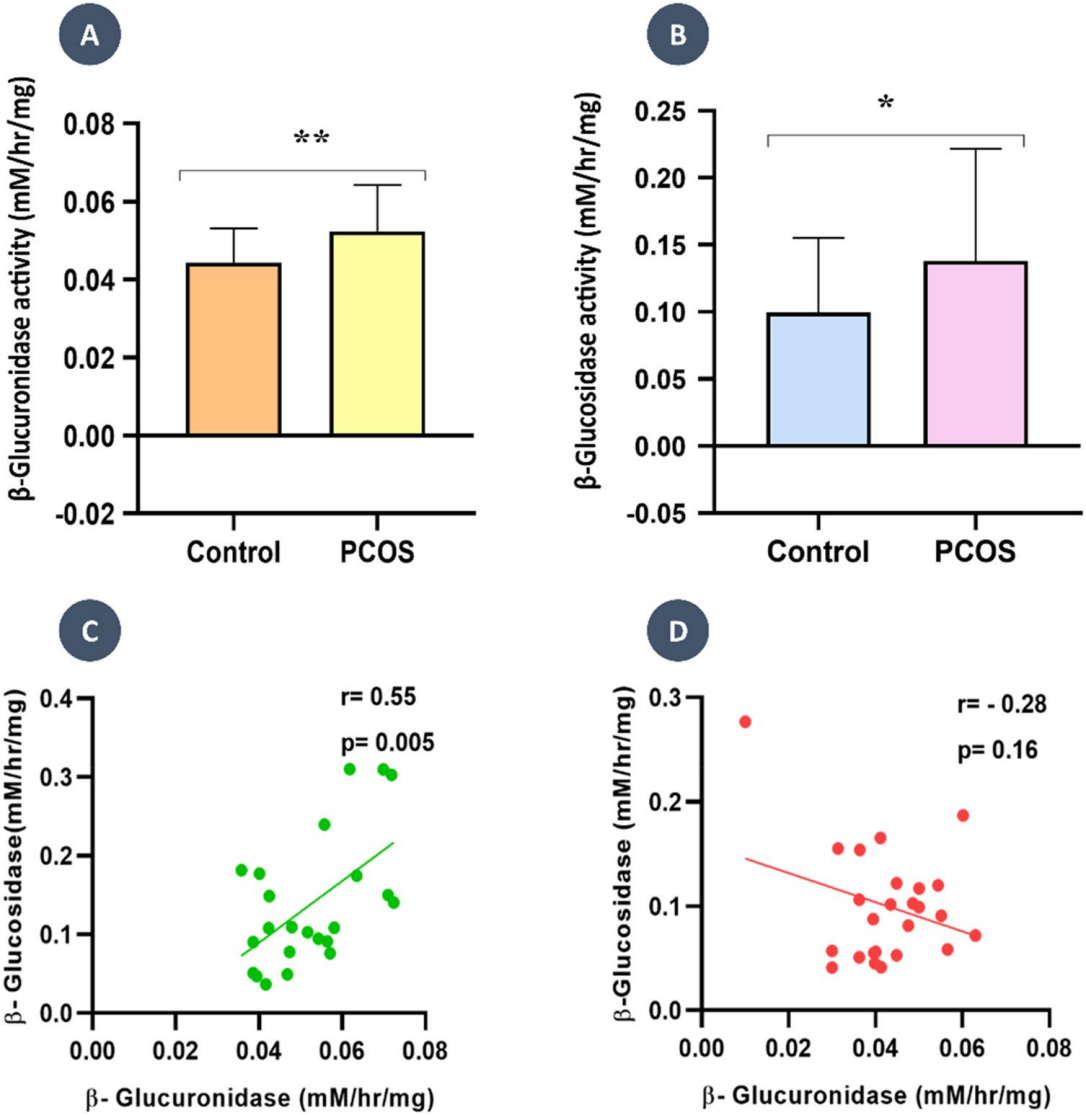
Average activity of Gut microbial enzymes and correlation analysis. (**A**) PCOS group present a higher level of gut microbial β-glucuronidase activity compared to Control groups with *p* < 0.05. (**B**) Gut microbial β-glucosidase activity shows high in the PCOS group compared to control groups with *p* > 0.05. (**C**) PCOS group shows a positive association between β-glucuronidase and β-glucosidase activities with r = 0.55 and *p* = 0.005. (**D**) The control group shows a negative association between β-glucuronidase and β-glucosidase activities with r = 0.16 and *p* = 0.16.

To understand the relationship between the gut microbial enzyme activity and PCOS fluctuations, the fecal β-glucuronidase and β-glucosidase activities of the women with PCOS and healthy control were measured. To determine the activity of β-glucuronidase and β-glucosidase from the fecal specimens, we used the regression formula obtained from the calibration curve of p-nitrophenol (PNP), i.e., y = 7.9262x + 0.0085 (Fig S2). Table 1 shows the average level of β-glucuronidase activity and β-glucosidase in PCOS and healthy control stool specimens. β-glucuronidase activity differed statistically significantly between the PCOS group and the controls (0.05 ± 0.01 vs. 0.04 ± 0.01, *p* = 0.01) (Fig. 2A). In contrast, β-glucosidase activity did not differ significantly between groups but was higher in the PCOS group (0.13 ± 0.08 vs. 0.09 ± 0.05, *p* = 0.06) (Fig. 2B).

### Correlation between β- glucuronidase and β- glucosidase activity

We were surprised to find that the activities of both enzymes were correlated. Therefore, we performed a Pearson correlation analysis of β- Glucuronidase activity and β-Glucosidase activity in each group. Interestingly, we found a statistically significant positive correlation between β-Glucuronidase activity and β-Glucosidase activity in the PCOS group (r = 0.55; *p* = 0.005 (Fig. 2C), Whereas in the controls, we found a negative correlation between β-Glucuronidase activity and β-Glucosidase activity (r = − 0.28; *p* = 0.16) (Fig. 2D), although it was not statistically significant.

### Correlation between gut microbial enzyme activity and serum sex hormone levels

**Figure 3:**
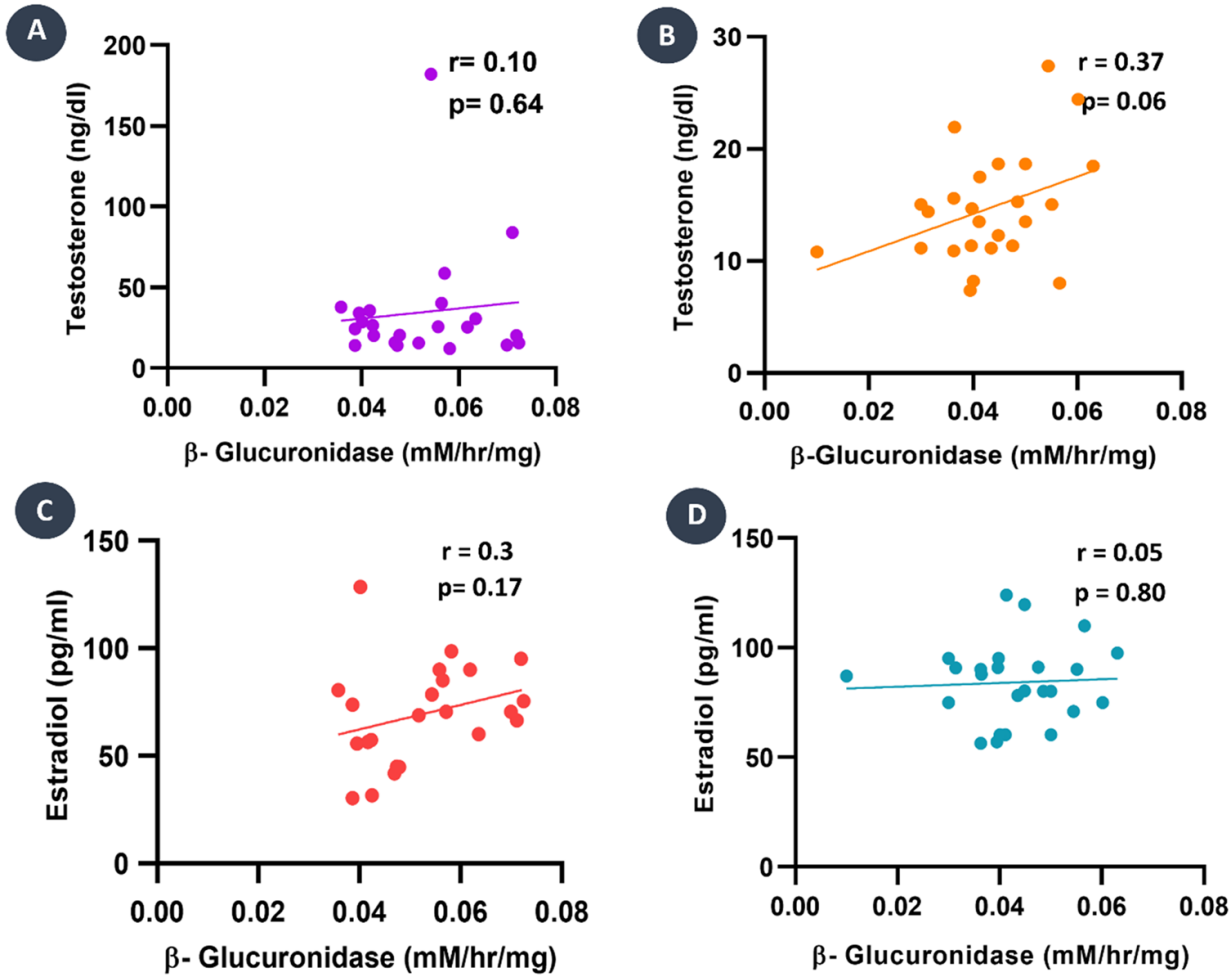
The correlation matrix of gut microbial β-glucuronidase activity with estradiol and testosterone levels. (**A**) PCOS group shows a positive association between testosterone level and β-glucuronidase activity with r = 0.10 and *p* = 0.64. (**B**) Control group shows a positive association between testosterone and β-glucuronidase activity with r = 0.37 and *p* = 0.06. (**C**) PCOS group shows a positive correlation between β-glucuronidase activity and estradiol with r = 0.3 and *p* = 0.17. (**D**) The control group shows a negligible positive correlation between β-glucuronidase activity and estradiol level with r = 0.05 and *p* = 0.80.

According to the Pearson correlation analysis of β-Glucuronidase activity with testosterone of PCOS and control groups, a moderate positive correlation existed in the PCOS while a strong positive association was found in the control group (r = 0.10, *p* = 0.64 vs. r = 0.37, *p* = 0.06 respectively) (Fig. 3A,B). However, it was not statistically significant. Wise, a positive correlation existed (r = 0.3, *p* = 0.17 vs. r = 0.05, *p* = 0.80 respectively) (Fig. 3C,D) in the PCOS group and no association was found in control with estradiol which is also statistically insignificant.

**Figure 4:**
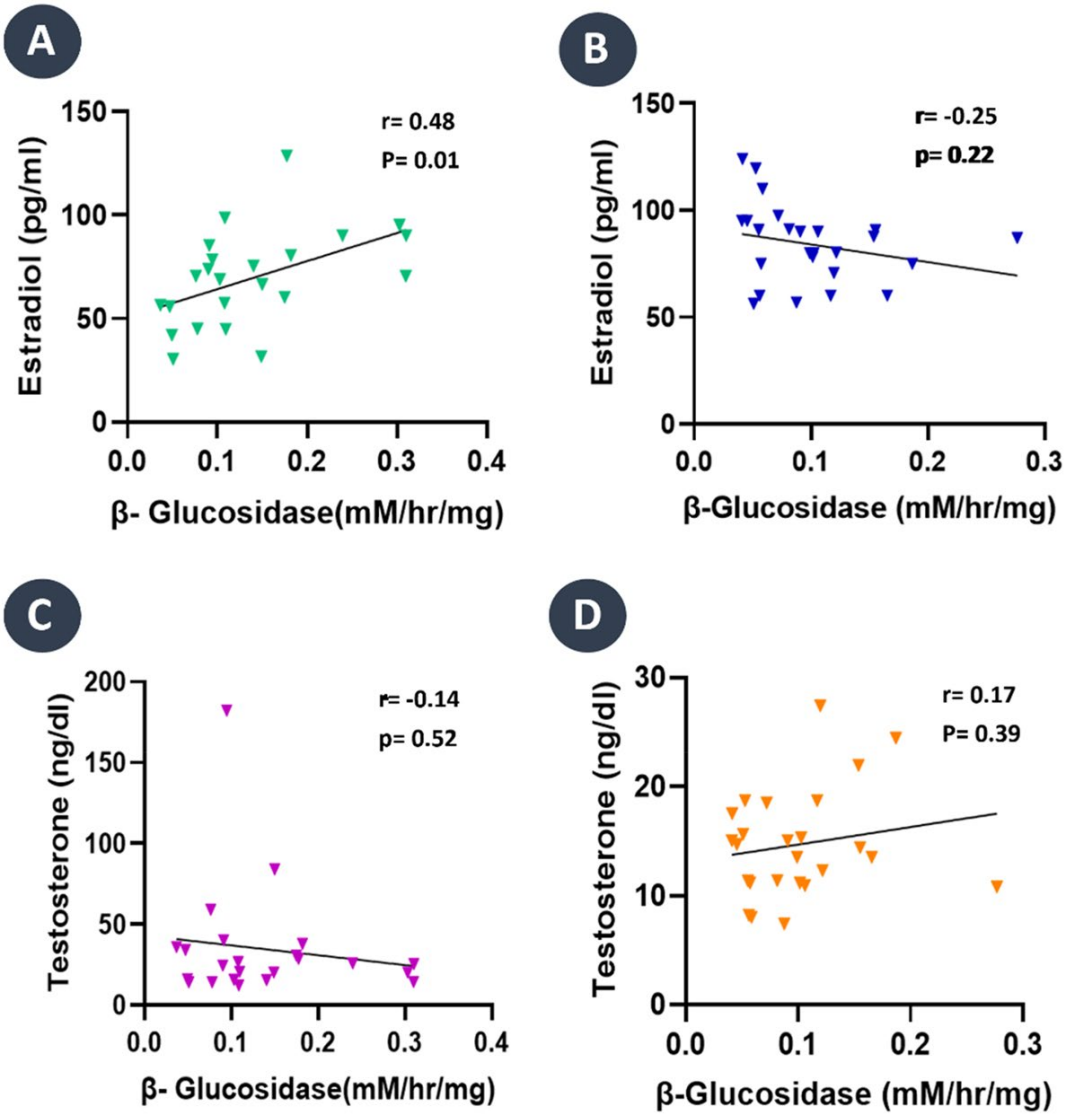
Correlation matrix of gut microbial β-glucosidase activity with estradiol and testosterone level. (**A**) PCOS group shows a positive correlation between β-glucosidase activity and estradiol with r = 0.48 and *p* = 0.01. (**B**) The control group shows a negative association between estradiol level and β-glucosidase activity with r = -0.25 and *p* = 0.22. (**C**) PCOS group shows a negative correlation between β-glucosidase activity and testosterone level r = − 0.14 and *p* = 0.52. (**D**) Control group shows a positive association between testosterone and β-glucosidase activity with r = 0.17 and *p* = 0.39.

The relationship between β-glucosidase activity with testosterone and estradiol in PCOS and the control group were examined. A statistically significant positive correlation between β-glucosidase activity and estradiol in the PCOS group (r = 0.48, *p* = 0.01) was observed (Fig. 4A). In contrast, a contradictory negative correlation (r = − 0.25, *p* = 0.22) was determined in control group (Fig. 4B) but it was not statistically significant. In the case of the association between β-glucosidase activity and testosterone, the study found a negative correlation in the PCOS group (r = − 0.14, *p* = 0.52) (Fig. 4C). In contrast, a positive correlation existed in controls (r = 0.17, p = 0.39) (Fig. 4D), although it was not statistically significant.

## Discussion

This study explored the distinctions in gut microbial β-glucuronidase and β-glucosidase activities between women with and without PCOS, which aimed to uncover the enzymes' impact on PCOS pathology.

PCOS is a complex endocrine disorder characterized by hormonal imbalances and hyperandrogenism. Similar to the previous report, elevated testosterone levels in the PCOS group were observed in the present study reaffirming the association between PCOS and hyperandrogenism^12^. In addition, high LH/FSH ratio was found in the selected women group of present work in agreement with the hormonal disruption reported in PCOS by Malini et al.^13^.

While investigating gut microbial enzymes' role in PCOS, a significant positive correlation was observed between β-glucuronidase and β-glucosidase activities, indicating the synergistic relationship of studied enzymes in PCOS conditions. However, random activities of both enzymes were found in the control group with normal physiological conditions, indicating a negative correlation.

The present work revealed the connections between the hormone and the activity of the selected enzymes where the testosterone was positively increased with an increase in β-glucuronidase in both groups. In a rodent study, β-glucuronidase from gut bacteria can alter androgen metabolism via deglucuronidation, potentially affecting systemic testosterone levels^14^. Additionally, results showed a moderate negative relationship between β-glucosidase and testosterone in women with PCOS, contrasting with a positive association in the control group. The observed negative correlation in the PCOS group indicates that higher testosterone levels may suppress the β-glucosidase activity, or the reduced β-glucosidase activity may contribute to the elevated testosterone level. In addition, these associations may be impacted by various other factors, such as the altered gut microbiome commonly seen in PCOS or changes in insulin sensitivity, which are known to influence testosterone levels and gut microbiota composition^15^. However, these contrasting correlations' underlying mechanisms and functional implications are yet to be elucidated. These intriguing patterns underscore the need for further research to understand the relationship between hormones and gut microbial enzymes and their role in PCOS.

We observed lower estradiol levels and significantly higher testosterone levels in PCOS women compared to the control group, as with the previous reports^16^. Moreover, there was a positive (statistically non-significant) correlation between estradiol levels and β-glucuronidase activity in both groups. The above observation aligns with the fact that β-glucuronidase allows estrogen to reenter the bloodstream, thereby increasing the pool of available estrogen^17^. The gut plays a vital role in expelling excess estrogen metabolites. Any disruption to the gut microbiota, a condition known as gut dysbiosis, can affect the activity of β-glucuronidase. Consequently, an increase in the presence of bacteria that produce β-glucuronidase may lead to elevated estrogen levels, and the elevation in estrogen may contribute to health conditions such as infertility, endometrial hyperplasia, endometriosis, and breast cancer^18,19^.

Furthermore, a significant positive correlation between β-glucosidase activity and estradiol was detected in the PCOS group, while a negative correlation was found in the control group. It indicates a complex interplay between hormones, particularly estradiol, and the activities of β-glucuronidase and β-glucosidase. The elevated activities of gut microbiome enzymes β-glucuronidase and β-glucosidase may influence the estrogen pool.

The present study reveals the importance of gut microbial enzymes in women with PCOS from urban regions of Gujarat (India) and highlights a complex interplay between enzyme and hormonal profiles. The gut-brain axis theory proposed that the effect of microbiome profile and sex hormones level may serve as the bridge between the central nervous system and endocrine system^20,21^. Karolina et al. reported a negative correlation between β -glucuronidase activity and androgen levels, and other metabolic parameters in overweight and obese targeted PCOS women only^22^. In contrast, the present study pioneered the correlation of gut microbial enzymes, namely β-glucuronidase and β-glucosidase in women with PCOS and control subjects with hormonal parameters. It has opened an exciting avenue for further exploration in the field of obesity and insulin resistance in gut microbiota in PCOS. The human gut microbiome is highly diverse, emphasizing the need for comprehensive clinical studies and extensive clinical cohorts to account for microbial diversity.

However, the study has limitations such as a small sample size, a cross-sectional design, and participant matching based on BMI, which may overlook other relevant factors. The lack of a metagenomic analysis and the absence of mechanistic data restrict the depth of our understanding of gut enzyme activities in PCOS. Nevertheless, our research offers valuable understanding of gut microbial enzyme profiles in PCOS women and the control group.

The gut microbial β-glucuronidase and β-glucosidase enzymes will be served as promising biomarkers for early detection and monitoring of PCOS in metabolically disturbed women. These findings would help suggest personalized probiotic treatment plans to PCOS women to restore gut microbiota and will pave the way for better therapeutic strategies. This groundbreaking report uncovers the highly significant correlation between enzymes and hormones in PCOS, offering crucial insights for preliminary investigations and clinical care.

## Supplementary Information

Supplementary Figures.

## Data Availability

The datasets used and analyzed during the current study are available from the corresponding author upon reasonable request.

## Abbreviations

PCOS: Polycystic ovary syndrome
NAFLD: Non-alcoholic fatty liver disease
BMI: Body mass index
WHR: Waist to hip ratio
FSH: Follicle stimulating hormone
LH: Luteinizing hormone
TSH: Thyroid stimulating hormone
DHEAS: Dehydroepiandrosterone sulfate
PRL: Prolactin
E2: Estradiol
T: Testosterone
PNP: P-nitrophenol
