# Supplementary material for: Assessment of gut microbial β-glucuronidase and β-glucosidase activity in women with polycystic ovary syndrome
Source: Sci Rep. 2023 Jul 24;13:11967. doi: 10.1038/s41598-023-39168-5 (PMC10366212; doi:10.1038/s41598-023-39168-5)
Supplement: Supplementary file 1 — Supplementary Figures. [file 41598_2023_39168_MOESM1_ESM.docx]

**Supplementary Data**


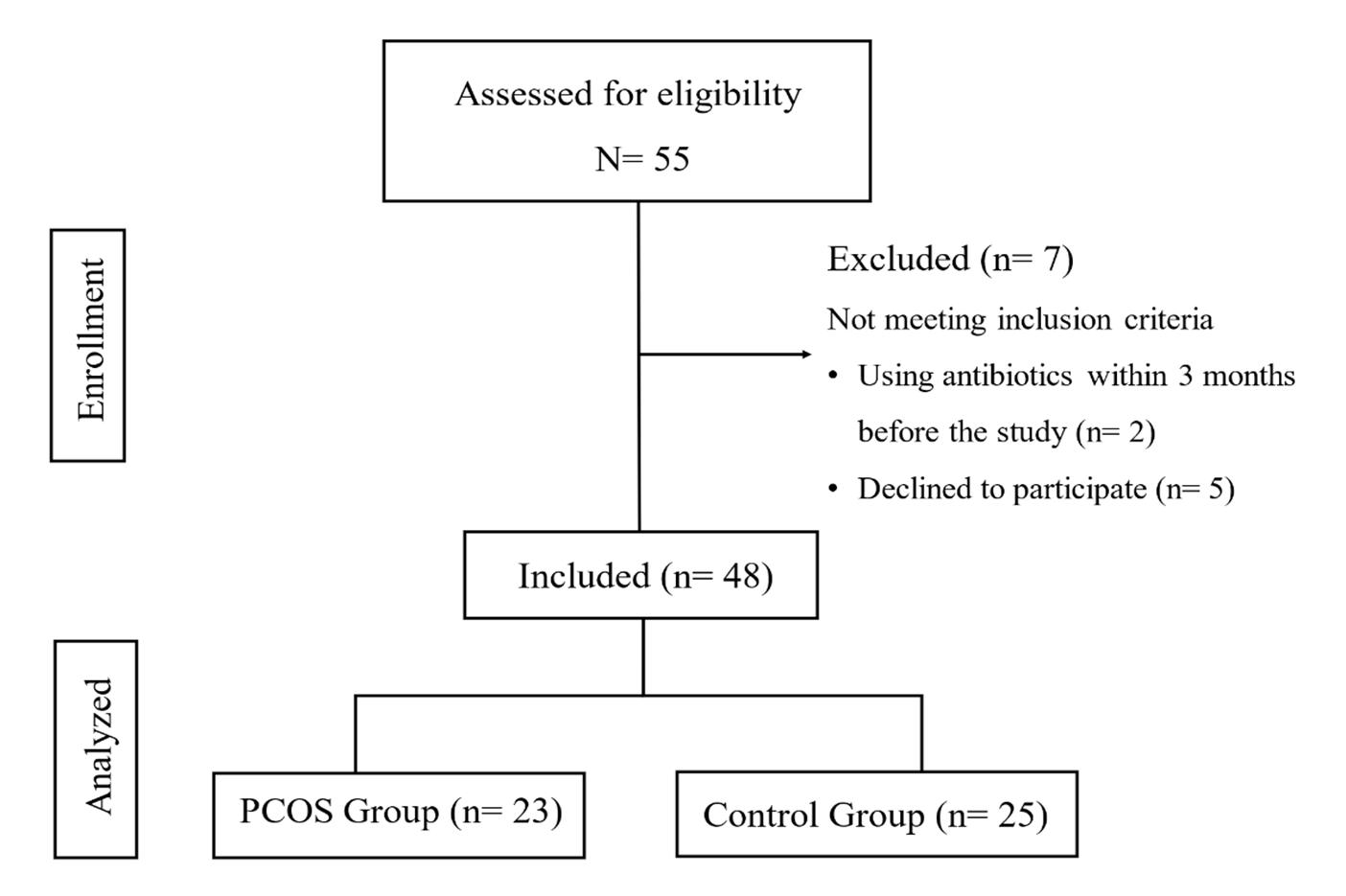


**Figure S1** Diagrammatic representation of participant recruitment in the study


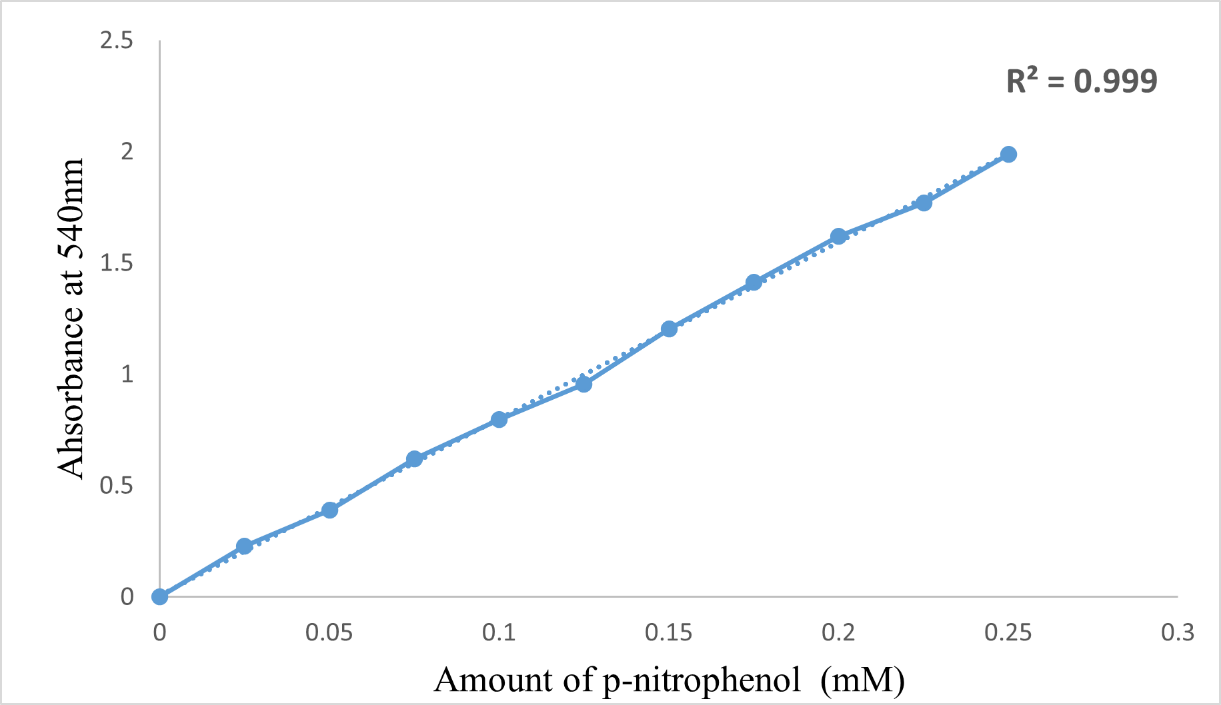


**Figure S2** Calibration curve of p-nitrophenol
